# The Prediction and Prognostic Significance of INPP5K Expression in Patients with Liver Cancer

**DOI:** 10.1155/2020/9519235

**Published:** 2020-04-25

**Authors:** Ruobing Wang, Yan Jiao, Yanqing Li, Siyang Ye, Guoqiang Pan, Shanshan Qin, Fang Hua, Yahui Liu

**Affiliations:** ^1^Department of Hepatobiliary and Pancreatic Surgery, The First Hospital of Jilin University, Changchun, Jilin 130021, China; ^2^Department of Pathophysiology, College of Basic Medical Sciences, Jilin University, Changchun, Jilin 130021, China; ^3^Department of Cardiology, The Second Hospital of Jilin University, Changchun, Jilin 130022, China; ^4^Department of Gastrointestinal Surgery, The First Hospital of Jilin University, Changchun, Jilin 130021, China; ^5^Department of Radiology, Affiliated Hospital of Qingdao University, Qingdao, Shandong 266000, China; ^6^Cardiovascular Internal Medicine, The First Hospital of Jilin University, Changchun, Jilin 130021, China

## Abstract

Liver cancer is a devastating disease for humans with poor prognosis. Although the survival rate of patients with liver cancer has improved in the past decades, the recurrence and metastasis of liver cancer are still obstacles for us. Inositol polyphosphate-5-phosphatase K (INPP5K) belongs to the family of phosphoinositide 5-phosphatases (PI 5-phosphatases), which have been reported to be associated with cell migration, polarity, adhesion, and cell invasion, especially in cancers. However, there have been few studies on the correlation of INPP5K and liver cancer. In this study, we explored the prognostic significance of INPP5K in liver cancer through bioinformatics analysis of data collected from The Cancer Genome Atlas (TCGA) database. Chi-square and Fisher exact tests were used to evaluate the relationship between INPP5K expression and clinical characteristics. Our results showed that low INPP5K expression was correlated with poor outcomes in liver cancer patients. Univariate and multivariate Cox analyses demonstrated that low INPP5K mRNA expression played a significant role in shortening overall survival (OS) and relapse-free survival (RFS), which might serve as the useful biomarker and prognostic factor for liver cancer. In conclusion, low INPP5K mRNA expression is an independent risk factor for poor prognosis in liver cancer.

## 1. Introduction

Hepatocellular carcinoma (HCC) is a devastating illness for humans, which has a poor prognosis with a relatively low 5-year survival rate [1]. Despite therapies for patients with HCC are improving now, the recurrence and metastasis of liver cancer are still unsurmountable obstacles for us. Moreover, it is a great challenge to predict the clinical outcomes for HCC patients. Thus, it is significant to find an effective screening strategy such as new specific markers to identify prognosis for patients.

INPP5K belongs to the family of PI 5- phosphatase, which catalyze the dephosphorylation of the phosphate of the inositol ring on position 5 [2, 3]. It is also known as skeletal muscle and kidney-enriched inositol phosphatase (SKIP) [4]. INPP5K regulates the actin cytoskeleton, myoblast differentiation and insulin signaling in skeletal muscle [4–6]. Homozygous deletion of INPP5K in mice results in embryonic lethality [6]. Literatures have reported that mutations in INPP5K were associated with congenital muscular dystrophies, cataract, and intellectual disability [5, 7]. Recently, PI 5- phosphatase has been reported to be associated with cell migration, polarity, adhesion, and cell invasion, especially in cancer cells. Moreover, the depletion of INPP5K may affect cell migration by the abundance of phosphatidylinositol 4,5-bisphosphate (PI(4,5)P2) under the stimulation of integrin [8]. The above research suggests the role of INPP5K in regulating the motility of cells, which is consistent with the characteristics of tumor metastasis and invasion.

However, the prognostic significance of INPP5K expression in liver cancer has not been reported yet. In this study, we intend to assess the independent prognostic value of INPP5K expression for overall survival of liver cancer patients. INPP5K expression in liver cancer was obtained from The Cancer Genome Atlas Liver Hepatocellular Carcinoma (TCGA-LIHC). Patients were divided into high and low INPP5K expression groups to explore the correlations between INPP5K expression and different clinical features of liver cancer. Our results indicated that INPP5K might be a biomarker for the diagnosis and prognosis of liver cancer.

## 2. Materials and Methods

### 2.1. Data Mining of TCGA Database

We collected the RNA-sequencing expression results (level three) from The Cancer Genome Atlas (TCGA) dataset by using R software (version 3.6.1) [9]. The pathological and clinical information of these patients was gathered following their ID in TCGA dataset. The patients' information included their ages, genders, histological grades, clinical stages, and T/N/M classifications. The INPP5K mRNA expression was evaluated as log2(*x* + 1) scores, which were converted to normalized RNA-Seq by Expectation-Maximization (RSEM) values for the further analysis.

### 2.2. Data Mining of ICGC Data Portal and GSE14520 Database

The gene expression results and clinical information of patients were also gathered from the International Cancer Genome Consortium (ICGC, https://icgc.org/) and Gene Expression Omnibus (GEO, https://www.ncbi.nlm.nih.gov/geo/) dataset. The GSE14520 microarray expression information was collected from GEO dataset. The results from TCGA dataset were verified by ICGC and GSE14520 dataset. These data were analyzed by R software.

### 2.3. Statistical Analyses

The expression of INPP5K in the TCGA-Liver Hepatocellular Carcinoma (LIHC) dataset was assessed using nonparametric rank sum tests and visualized in box plots. The Wilcoxon rank sum test was performed to analyze the differences between two subgroups, including the gender, age, and vital status. The Kruskal-Wallis test was performed to analyze the differences in three or more subgroups, including the histologic grades, clinical stages, and T/N/M classifications. To explore the relationship between INPP5K mRNA expression and clinical information, we drew the receiver operating characteristic (ROC) curve by using the pROC package [10]. According to the AUC value identified from the ROC curve, the samples were divided into high/low INPP5K mRNA expression groups. The correlation between clinical parameters and high/low INPP5K mRNA expression was conducted by Chi-square tests and Fisher exact tests. Kaplan-Meier analysis was performed to compare the differences in OS and RFS between high/low INPP5K mRNA expression groups based on the log-rank test using the survival package in R [11, 12]. Univariate Cox regression analysis was performed to select the potential prognostic factors by calculating the hazard ratios (HRs) and 95 percent confidence intervals (CIs). Multivariate Cox regression analysis was used to verify the correlation of INPP5K mRNA expression with OS and RFS, along with the clinical parameters which were associated with clinical prognosis in univariate analysis. Statistical analysis was conducted with the R software (version 3.6.1). ^∗^*P* < 0.05 was considered as significant.

## 3. Result

### 3.1. Patient Characteristics

Clinical information of 373 patients with liver cancer was downloaded and organized from The Cancer Genome Atlas Liver Hepatocellular Carcinoma (TCGA-LIHC), including age, gender, histological type, histologic grade, TNM classification, radiation therapy, residual tumor status, vital status, sample type, overall survival, and relapse-free survival (Table 1).

### 3.2. Low INPP5K Expression and Its Diagnostic Value in Liver Cancer

**Figure 1:**
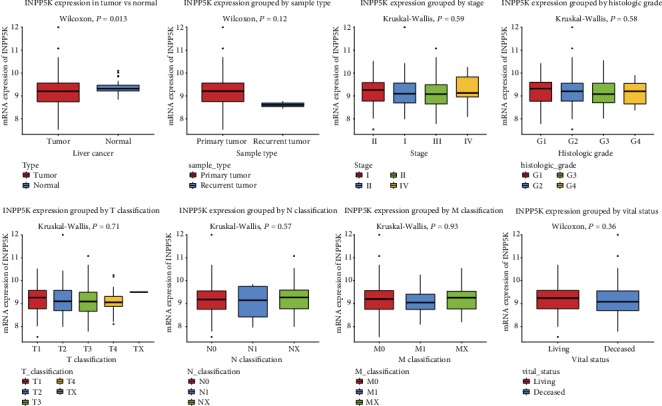
INPP5K expression in liver cancer. INPP5K expression of liver cancer tissues was compared with that in normal according to age, gender, histological type, histologic grade, TNM classification, radiation therapy, residual tumor status, vital status, sample type, overall survival, and relapse-free survival.

We compared the expression of INPP5K mRNA in liver cancer (*n* = 373) and normal liver (*n* = 50) tissue via box plots (Figure 1). The results indicated that INPP5K expression was lower in liver cancer (*P* = 0.013). Consistent with the results in the TCGA cohort, the mRNA expression of INPP5K was significantly downregulated in liver cancer tissues (*n* = 225) when compared with normal liver tissues (*n* = 220) in GSE14520 cohort (*P* ≤ 0.0001; Supplementary Fig. S1).

**Figure 2:**
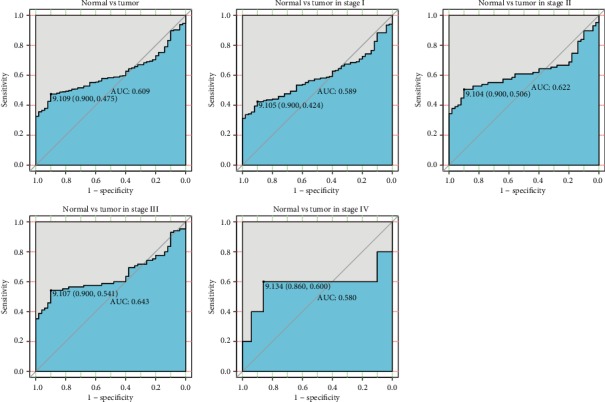
The diagnosis value of INPP5K mRNA expression in liver cancer. The ROC curve for INPP5K expression of liver cancer tissues was compared with that in normal. Subgroup analysis for stages I, II, III, and IV liver cancer.

The ROC curve was performed using the expression data from TCGA-LIHC to evaluate the diagnostic value of INPP5K (Figure 2(a)). The area under the ROC curve (AUC) was 0.609, which showed the modest diagnostic value of INPP5K. Subgroup analysis manifested the diagnostic value in different stages of liver cancer with the AUC values of 0.589, 0.622, 0.643, and 0.580 for stage I, stage II, stage III, and stage IV, respectively (Figures 2(b)–2(e)).

### 3.3. Correlations between Clinical Features and INPP5K Expression in Liver Cancer

To assess the correlations between INPP5K expression and clinical features of liver cancer, patients were divided into high and low INPP5K mRNA expression groups according to the threshold determined by ROC curve (Table 2). The Chi-square test showed that low INPP5K expression was significantly correlated with survival status (*P* = 0.0276), overall survival (*P* = 0.0236), and relapse-free survival (*P* = 0.0004).

### 3.4. Low INPP5K Expression Is an Independent Risk Factor for Overall Survival in Liver Cancer Patients

**Figure 3:**
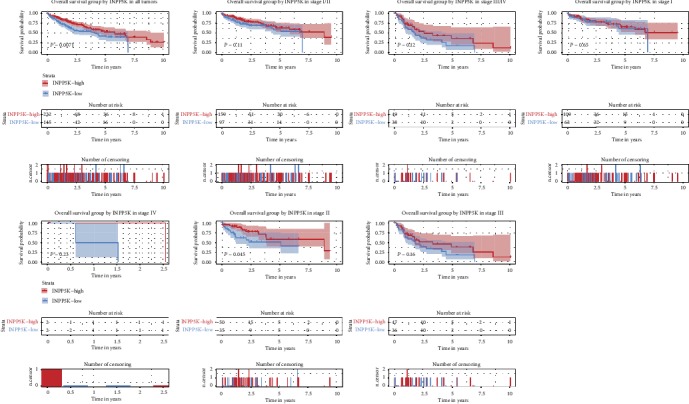
Kaplan-Meier curves for OS according to INPP5K expression in liver cancer. Survival analysis and subgroup analysis according to clinical stage were performed based on Kaplan-Meier curves.

To evaluate the diagnostic value of INPP5K in liver cancer patients, Kaplan-Meier survival curve with the log-rank test was executed, which indicated that low INPP5K expression was associated with poor overall survival (*P* = 0.0071; Figure 3). Subgroup analysis showed that low INPP5K expression was correlated with poor overall survival of cases with clinical stage II (*P* = 0.045; Figure 3). Consistent with the results in the TCGA cohort, the validation of survival analysis was conducted by ICGC cohort (*P* = 0.025; Supplementary Fig. S2).

Both univariate and multivariate Cox analyses showed that the expressions of INPP5K (hazard ratio = 1.5, 95% confidence interval: 1.06-2.13, *P* = 0.023), residual tumor (hazard ratio = 1.42, 95% confidence interval: 1.11-1.82, *P* = 0.005), and T classification (hazard ratio = 1.81, 95% confidence interval: 1.44-2.29, *P* ≤ 0.001; Table 3) were independent risk factors for poor overall survival in liver cancer patients.

### 3.5. Low INPP5K Expression Is an Independent Risk Factor for Relapse-Free Survival in Liver Cancer Patients

**Figure 4:**
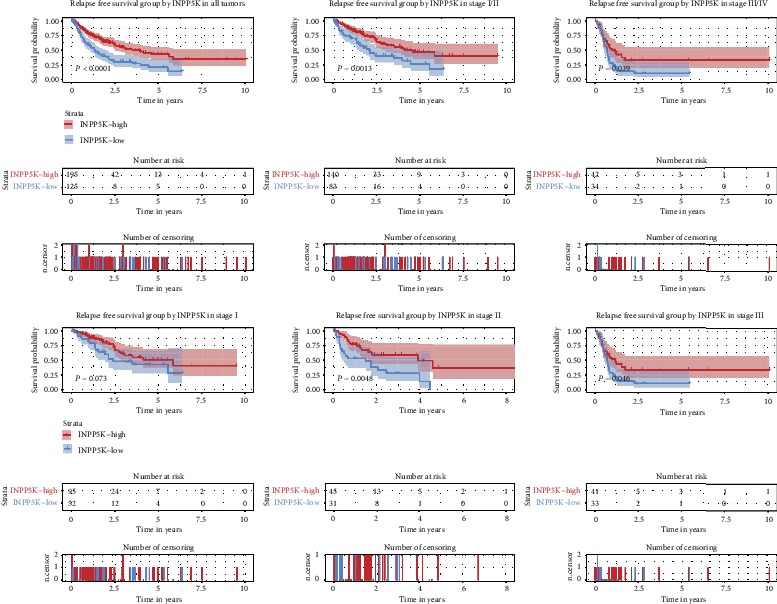
Kaplan-Meier curves for RFS according to INPP5K expression in liver cancer. Survival analysis and subgroup analysis according to clinical stage were performed based on Kaplan-Meier curves.

To evaluate the diagnostic value of INPP5K in liver cancer patients, Kaplan-Meier survival curve with the log-rank test was executed, which indicated that low INPP5K expression was associated with relapse-free survival (*P* ≤ 0.0001; Figure 4). Subgroup analysis showed that low INPP5K expression was correlated with poor RFS of cases with clinical stage I/II (*P* = 0.0013; Figure 4) and stage III/IV (*P* = 0.039; Figure 4).

Univariate and multivariate Cox analyses showed that the expression of INPP5K (hazard ratio = 2.03, 95% confidence interval: 1.45-2.83, *P* ≤ 0.001), residual tumor (hazard ratio = 1.3, 95% confidence interval: 1.02-1.66, *P* = 0.035), and T classification (hazard ratio = 1.66, 95% confidence interval: 1.27-2.15, *P* ≤ 0.001; Table 4) were independent risk factors for RFS in liver cancer patients.

## 4. Discussion

Liver cancer is associated with a high mortality rate worldwide. Despite the rapid development of medicine, the recurrence and metastasis of liver cancer remain unsolvable. Prognostic markers have numerous potential roles in cancer. They can help to predict patients' outcomes to improve clinical decision-making and screen patients who are most likely to respond to the particular treatment. Therefore, it is important to find reliable biomarkers for diagnosis and prognosis in liver cancer. In recent years, bioinformatics has attracted much attention because of its significance in screening markers. We also have been working on the exploration of tumor biomarkers by bioinformatics [13–21].

In previous studies, some prognostic biomarkers of liver cancers have been identified through bioinformatics. The high mRNA expression of pescadillo (PES1), high mobility group A2 (HMGA2), microtubule-associated serine and threonine kinase 2 (MAST2), trophinin-associated protein (TROAP), and MEX3A was associated with poor prognosis for liver cancer [18, 22–25]. Among these biomarkers, MAST2, PES1, and HMGA2 are involved in the regulation of chromosomal instability, DNA replication, cell proliferation, and cell cycle progression. Meanwhile, TROAP and MEX3A are related to the adhesion and cell migration. On the contrary, the low mRNA expression of oxoglutarate dehydrogenase-like (OGDHL) and phosphoglucomutase-like protein 5 (PGM5) was associated with poor prognosis for liver cancer [16, 26]. These two biomarkers, acting as the putative tumor suppressor genes, play prominent roles in regulating the metabolic reprogramming process in cancers.

In this study, we found that INPP5K was lowly expressed in liver cancer and low expression of the INPP5K mRNA was associated with poor survival status and recurrence in liver cancer. Low INPP5K expression was correlated poor outcomes in liver cancer patients by the Chi-square test. Univariate and multivariate Cox analyses demonstrated that INPP5K mRNA expression played a significant role in overall survival and relapse-free survival, which might be a useful biomarker and prognostic factor for liver cancer. The diagnostic value of INPP5K expression was also confirmed by Kaplan-Meier curves with the log-rank test for OS and RFS.

PI(4,5)P2, a multifunctional lipid, is essential for regulating several basic subcellular processes in eukaryotic cells. It is also a key lipid messenger that regulates cell migration. Recently, the link between the dynamic balance of PI(4,5)P2 and the mechanisms driving cell polarity and migration has been reported. It participates in the cytoskeletal organization via regulating related proteins (Mena, Tks5, or lamellipodin) [27]. Moreover, PI(4,5)P2 is involved in dynamic focal adhesion complexes and controls the migration and invasion of various cancer cells [28–30].

INPP5K is one of the PI 5-phosphatases, which catalyze the substrates such as PI(4,5)P2 and PI(3,4)P2 [2]. Similarly like the PI(4,5)P2, PI 5-phosphatases also have been reported to regulate cell migration and invasion in cancer cells. In glioblastoma, the decrease of SH2 domain-containing inositol 5-phosphatase 2 (SHIP2) expression may have a positive or negative effect on cell migration rates depending on the types [31, 32]. In addition, PI 5-phosphatase SHIP2 or INPP5K can be located on the plasma membrane and reduce the abundance of PI(3,4,5)P3 or PI(4,5)P2 [8]. Therefore, the association of low INPP5K expression with poor survival in liver cancer patients may be due to the effect of INPP5K on cell migration and invasion by controlling the abundance of P I(4,5)P2. Besides, our results showed that low INPP5K expression correlated significantly with poor RFS in liver cancer cases of nearly all clinical stage except the stage I, which suggested that INPP5K might not regulate the migration at the initiation of tumor formation. As the expression of INPP5K was lower in the deceased than in the living, the relationship between INPP5K and survival needs to be further explored.

Gene expression and genetic characteristics of tumor are relevant to clinical features, pathological features, and the prognosis of the patients. Data from genomic profiling suggested there are two major molecular clusters (proliferation and nonproliferation) in liver cancer with differential enrichment in prognostic features and the activation of signaling pathway. In the proliferation signaling pathway, the expression of several biomarkers, namely, NOTCH, TGF-*β* proteins, and several genes, is primarily associated with poor prognosis of patients [33]. It has been reported that the signature of 186 genes in liver cancer surrounding tissues can predict the higher risk of tumor recurrence after resection for liver cancer patients [34]. And these results are also associated with outcomes of patients with hepatitis C-related early-stage cirrhosis [35]. In addition, some clinical trials are being conducted based on the high MET expression and the mutations of RAS in tumor cells to find the potential biomarkers for predicting advanced HCC [36]. These studies suggest the feasibility of conducting tissue biomarker studies in patients with liver cancer. Therefore, the genetic biomarkers correlated with the prognosis of liver cancer should be more widely validated in clinical trials.

This is the first study that suggested the relationship between INPP5K and clinical characteristics in liver cancer patients by mining TCGA database so far, which indicated that low INPP5K mRNA expression may serve as an independent prognostic factor for poor survival in liver cancer. However, due to sample size limitation, it is difficult to establish a predicting model for INPP5K expression and clinicopathological variables in liver cancer. In further study, we will expand the sample size to explore the prognostic value of INPP5K expression and build an appropriate predicting model for the prognosis of liver cancer patients.

## 5. Conclusions

In this study, we assessed the independent prognostic value of INPP5K expression by mining TCGA database. Our results demonstrated that INPP5K was lowly expressed in liver cancer. The decreased expression level of INPP5K was related to poor prognosis, which could act as an independent risk factor for OS and RFS in liver cancer patients. This finding identified that low INPP5K expression was an independent factor involved in the prognosis of liver cancer and associated with poor survival.

## Figures and Tables

**Table 1:** Clinical characteristics of the liver cancer patients.

Characteristics	Numbers of cases (%)
Age	
<55	117 (31.45)
≥55	255 (68.55)
Gender	
Female	121 (32.44)
Male	252 (67.56)
Histological type	
Fibrolamellar carcinoma	3 (0.8)
Hepatocellular carcinoma	363 (97.32)
Hepatocholangiocarcinoma (mixed)	7 (1.88)
Histologic grade	
NA	5 (1.34)
G1	55 (14.75)
G2	178 (47.72)
G3	123 (32.98)
G4	12 (3.22)
Stage	
NA	24 (6.43)
I	172 (46.11)
II	87 (23.32)
III	85 (22.79)
IV	5 (1.34)
T classification	
NA	2 (0.54)
T1	182 (48.79)
T2	95 (25.47)
T3	80 (21.45)
T4	13 (3.49)
TX	1 (0.27)
N classification	
NA	1 (0.27)
N0	253 (67.83)
N1	4 (1.07)
NX	115 (30.83)
M classification	
M0	267 (71.58)
M1	4 (1.07)
MX	102 (27.35)
Radiation therapy	
NA	25 (6.7)
No	340 (91.15)
Yes	8 (2.14)
Residual tumor	
NA	7 (1.88)
R0	326 (87.4)
R1	17 (4.56)
R2	1 (0.27)
RX	22 (5.9)
Vital status	
Deceased	130 (34.85)
Living	243 (65.15)
Sample type	
Primary tumor	371 (99.46)
Recurrent tumor	2 (0.54)
Os_s	
0	237 (64.58)
1	130 (35.42)
Rf_s	
0	179 (55.94)
1	141 (44.06)
INPP5K	
High	225 (60.32)
Low	148 (39.68)
Type	
1	373 (100)

**Table 2:** Relationship between the clinical features of liver cancer and INPP5K expression.

Parameter	Variables	*N*	High	%	Low	%	X2	*P*	Fish
Age	<55	117	66	(29.46)	51	(34.46)	0.8127	0.3673	0.3615
	≥55	255	158	(70.54)	97	(65.54)			
Gender	Female	121	75	(33.33)	46	(31.08)	0.1166	0.7327	0.7346
	Male	252	150	(66.67)	102	(68.92)			
Histological type	Fibrolamellar carcinoma	3	3	(1.33)	0	(0)	3.9605	0.138	0.1695
	Hepatocellular carcinoma	363	216	(96)	147	(99.32)			
	Hepatocholangiocarcinoma (mixed)	7	6	(2.67)	1	(0.68)			
Histologic grade	G1	55	38	(17.19)	17	(11.56)	4.21	0.2397	0.2344
	G2	178	110	(49.77)	68	(46.26)			
	G3	123	66	(29.86)	57	(38.78)			
	G4	12	7	(3.17)	5	(3.4)			
Stage	I	172	109	(51.66)	63	(45.65)	1.2996	0.7292	0.7014
	II	87	51	(24.17)	36	(26.09)			
	III	85	48	(22.75)	37	(26.81)			
	IV	5	3	(1.42)	2	(1.45)			
T classification	T1	182	115	(51.57)	67	(45.27)	2.2864	0.6832	0.7163
	T2	95	55	(24.66)	40	(27.03)			
	T3	80	45	(20.18)	35	(23.65)			
	T4	13	7	(3.14)	6	(4.05)			
	TX	1	1	(0.45)	0	(0)			
N classification	N0	253	150	(66.96)	103	(69.59)	0.5399	0.7634	0.6991
	N1	4	2	(0.89)	2	(1.35)			
	NX	115	72	(32.14)	43	(29.05)			
M classification	M0	267	159	(70.67)	108	(72.97)	0.4947	0.7809	0.7461
	M1	4	2	(0.89)	2	(1.35)			
	MX	102	64	(28.44)	38	(25.68)			
Radiation therapy	No	340	208	(97.65)	132	(97.78)	0	1	1
	Yes	8	5	(2.35)	3	(2.22)			
Residual tumor	R0	326	198	(90.41)	128	(87.07)	2.7596	0.4302	0.4237
	R1	17	8	(3.65)	9	(6.12)			
	R2	1	0	(0)	1	(0.68)			
	RX	22	13	(5.94)	9	(6.12)			
Vital status	Deceased	130	68	(30.22)	62	(41.89)	4.8529	**0.0276**	**0.0262**
	Living	243	157	(69.78)	86	(58.11)			
Sample type	Primary tumor	371	225	(100)	146	(98.65)	1.0481	0.3059	0.1568
	Recurrent tumor	2	0	(0)	2	(1.35)			
Os_s	0	237	154	(69.37)	83	(57.24)	5.1222	**0.0236**	**0.0193**
	1	130	68	(30.63)	62	(42.76)			
Rf_s	0	179	125	(64.1)	54	(43.2)	12.668	**0.0004**	**0.0003**
	1	141	70	(35.9)	71	(56.8)			

**Table 3:** Univariate and multivariate analyses of the correlation of INPP5K expression with OS among liver cancer patients.

Parameters	Univariate analysis			Multivariate analysis		
	Hazard.Ratio.x	CI95.x	Pvalue.x	Hazard.Ratio.y	CI95.y	Pvalue.y
Age	1.00	0.69-1.45	0.997			
Gender	0.80	0.56-1.14	0.220			
Histological type	0.99	0.27-3.66	0.986			
Histologic grade	1.04	0.84-1.30	0.698			
Stage	1.38	1.15-1.66	**0.001**	0.87	0.70-1.08	0.205
T classification	1.66	1.39-1.99	**0.001**	1.81	1.44-2.29	**0.001**
N classification	0.73	0.51-1.05	0.086			
M classification	0.72	0.49-1.04	0.077			
Radiation therapy	0.51	0.26-1.03	0.060			
Residual tumor	1.42	1.13-1.80	**0.003**	1.42	1.11-1.82	**0.005**
INPP5K	1.61	1.13-2.28	**0.008**	1.50	1.06-2.13	**0.023**

**Table 4:** Univariate and multivariate analyses of the correlation of INPP5K expression with RFS among liver cancer patients.

Parameters	Univariate analysis			Multivariate analysis		
	Hazard.Ratio.x	CI95.x	Pvalue.x	Hazard.Ratio.y	CI95.y	Pvalue.y
Age	0.90	0.63-1.28	0.550			
Gender	0.99	0.70-1.41	0.966			
Histological type	2.02	0.66-6.24	0.220			
Histologic grade	0.98	0.80-1.21	0.883			
Stage	1.66	1.38-1.99	**0.001**	1.11	0.86-1.43	0.416
T classification	1.78	1.49-2.12	**0.001**	1.66	1.27-2.15	**0.001**
N classification	0.97	0.67-1.40	0.874			
M classification	1.17	0.79-1.74	0.432			
Radiation therapy	0.74	0.26-2.16	0.584			
Residual tumor	1.28	1.01-1.61	**0.042**	1.30	1.02-1.66	**0.035**
INPP5K	2.09	1.50-2.90	**0.001**	2.03	1.45-2.83	**0.001**

## Data Availability

The data used to support the findings of this study are included within the article.

## Supplementary Materials

Supplementary Materials Figure legends: Supplementary Figure S1: the INPP5K mRNA expression in the GSE14520 cohort. The validation of INPP5K mRNA expression in liver cancer and normal liver tissues was conducted by GSE14520 cohort. Supplementary Figure S2: Kaplan-Meier curves for OS according to INPP5K expression in the ICGC cohort. The validation of survival analysis was conducted by ICGC cohort.
