# Supplementary material for: The Prediction and Prognostic Significance of INPP5K Expression in Patients with Liver Cancer
Source: Biomed Res Int. 2020 Apr 25;2020:9519235. doi: 10.1155/2020/9519235 (PMC7201693; doi:10.1155/2020/9519235)

Figure legends

Supplementary Fig. S1 The INPP5K mRNA expression in the GSE14520 cohort.

The validation of INPP5K mRNA expression in liver cancer and normal liver tissues was conducted by GSE14520 cohort.

Supplementary Fig. S2 Kaplan-Meier curves for OS according to INPP5K expression in the ICGC cohort.

The validation of survival analysis was conducted by ICGC cohort.

Supplementary Fig. S1

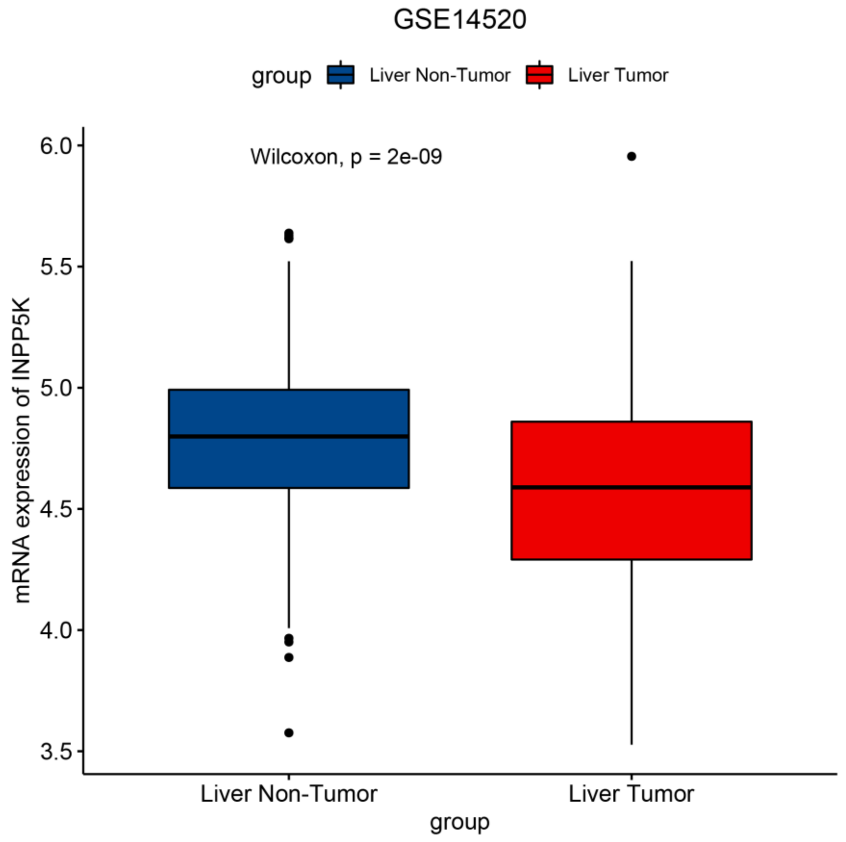

Supplementary Fig. S2

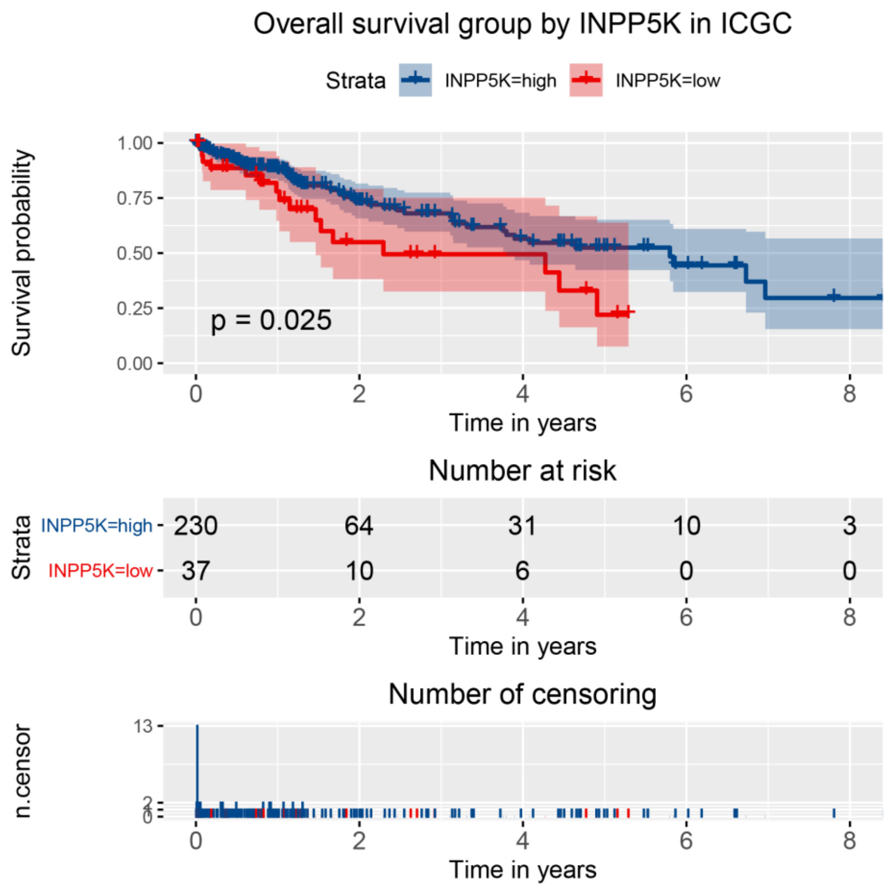

Supplement: Supplementary Materials — Figure legends: Supplementary Figure S1: the INPP5K mRNA expression in the GSE14520 cohort. The validation of INPP5K mRNA expression in liver cancer and normal liver tissues was conducted by GSE14520 cohort. Supplementary Figure S2: Kaplan-Meier curves for OS according to INPP5K expression in the ICGC cohort. The validation of survival analysis was conducted by ICGC cohort. [file 9519235.f1.pdf]
